# Potential Benefits of Anthocyanins in Chronic Disorders of the Central Nervous System

**DOI:** 10.3390/molecules28010080

**Published:** 2022-12-22

**Authors:** Sunil K. Panchal, Lindsay Brown

**Affiliations:** 1 School of Science, Western Sydney University, Richmond, NSW 2753, Australia; 2 School of Pharmacy and Medical Science, Griffith University, Gold Coast, QLD 4222, Australia

**Keywords:** anthocyanins, Alzheimer’s, Parkinson’s, autism, anxiety, gut–brain axis

## Abstract

Anthocyanins have been shown to be effective in chronic diseases because of their antioxidant and anti-inflammatory effects together with changes in the gut microbiota and modulation of neuropeptides such as insulin-like growth factor-1. This review will examine whether these mechanisms may be effective to moderate the symptoms of disorders of the central nervous system in humans, including schizophrenia, Parkinson’s disease, Alzheimer’s disease, autism spectrum disorder, depression, anxiety, attention-deficit hyperactivity disorder and epilepsy. Thus, anthocyanins from fruits and berries should be considered as complementary interventions to improve these chronic disorders.

## 1. Introduction

The Global Burden of Diseases, Injuries and Risk Factors Study 2019 presented the global, regional and national prevalence of depressive disorders, anxiety disorders, bipolar disorder, schizophrenia, autism spectrum disorders, conduct disorder, attention-deficit hyperactivity disorder, eating disorders, idiopathic developmental intellectual disability and a residual category of other central nervous system disorders from 1990 to 2019 [1]. This extensive study showed that central nervous system disorders remained among the top ten causes of disease burden with the proportion of disability-related life years from central nervous system disorders increasing from 3.1 to 4.9%. The study recommends that the delivery of effective prevention and treatment programmes is imperative.

**Figure 1:**
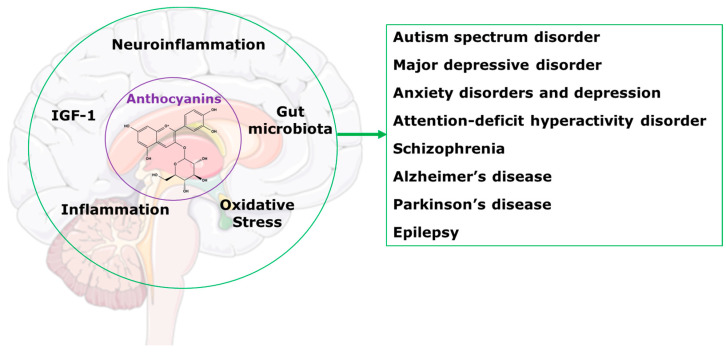
Potential mechanisms for anthocyanins in the central nervous system disorders.

This review will present information indicating that treatment with anthocyanins could be an effective programme to improve the health of people with central nervous system disorders. We have previously reviewed the benefits of anthocyanins as treatments for a wide range of chronic human diseases [2]. We showed that health benefits included reduced cognitive decline; protection of organs such as the liver, as well as the cardiovascular system, gastrointestinal tract and kidneys; improvements in bone health and obesity; and regulation of glucose and lipid metabolism. We also reviewed the most likely mechanisms of these improvements as alterations in gut microbiota, reduced oxidative stress and inflammation, and modulation of neuropeptides such as insulin-like growth factor-1 (Figure 1) [2]. This review focusses on the applications of these mechanisms to chronic diseases of the central nervous system.

## 2. Anthocyanins and Pharmacokinetics

Anthocyanins are secondary plant metabolites distributed in many fruits and vegetables as purple, blue, pink and red-coloured compounds with important nutritional value and health effects [3]. Recently, we highlighted dietary sources of anthocyanins and some of the advanced methods to obtain anthocyanins from agri-waste [2]. We discussed important mechanisms of actions of anthocyanins in improving chronic diseases including modulating the gut microbiota, decreasing oxidative stress and inflammation, and increasing insulin-like growth factor 1, and then summarised the therapeutic responses to anthocyanins in chronic human diseases [2]. Our current aim is to examine whether there are commonalities between the mechanisms of action of the anthocyanins and the known physiological and biochemical changes in central nervous system disorders. If so, the inference should be considered as plausible that anthocyanins have the potential to improve the chronic symptoms of the central nervous system disorders.

While anthocyanins are usually consumed as part of the diet, therapeutic studies will require high-purity compounds. Improved extraction and purification methods are being developed to meet these needs [4]. Strategies include efficient green extraction with ultrasound, pulsed electric fields, supercritical carbon dioxide and high pressure extraction as well as the semi-synthesis or de novo synthesis by microorganisms [5].

The pharmacokinetics of anthocyanins from food are complex, leading to a broad range of metabolites which may have biological activity [6]. After ingestion, anthocyanins from fruits and vegetables are partially absorbed (~35%) in the upper gastrointestinal tract while most of the anthocyanins (~65%) pass into the colon [7]. After absorption, dietary anthocyanins are metabolised to glucuronides, sulphates and methylates in the intestinal epithelium, liver and kidneys [7]. The major site of degradation of anthocyanins is the colon where the gut microbiota hydrolyse the glycosylated forms and cleave the anthocyanin heterocycle forming benzoic acids and phloroglucinol derivatives [8] which may be absorbed contributing to the bioavailable anthocyanin metabolites [6]. Gut microbiota-derived metabolites of anthocyanins include phloroglucinol derivatives, 4-hydroxybenzoic acid, protocatechuic acid, gallic acid, vanillic acid, syringic acid, catechol, pyrogallol, resorcinol, tyrosol, 3-(3′-hydroxyphenyl) propionic acid, dihydrocaffeic acid, 3-(4′-hydroxyphenyl) lactic acid, ferulic acid and hippuric acid [7].

A study using ^13^C-labelled anthocyanins in humans identified pharmacokinetic profiles of 17 metabolites in the circulation, 31 metabolites in the urine and 28 metabolites in faeces [9]. Parent anthocyanin represented only ~2% of the total metabolites in the circulation suggesting that responses to anthocyanins are likely mediated by its intermediates [9]. Anthocyanin metabolism is expected to differ among populations based on inter- or intra-individual variability; factors responsible for this variation include food matrix, food processing, genetic factors determining enzymatic levels, diet, age, sex and gut microbiota functionality [10]. Tryptophan and kynurenic acid were modulated by anthocyanins from blackberry in high fat diet-fed rats suggesting their roles in anti-neuroinflammatory pathways without affecting kynurenine [11].

The complex pharmacokinetics of anthocyanins makes it difficult to predict if the physiological responses are due to original compounds or their metabolites. Further, it is not known what the optimal doses would be for the anthocyanins due to their lower absorption and increased metabolism.

## 3. Anthocyanins in Central Nervous System Disorders

Since anthocyanins alter the gut microbiota, show anti-inflammatory and anti-oxidative responses and modify some brain neuropeptides, this section will examine how these mechanisms could provide therapeutic actions to prevent or attenuate brain disorders in humans.

Ageing is a remarkably complex process but some molecular pathways may influence health span in humans [12]. While calorie restriction increases lifespan in some species and overeating and obesity shortens lifespan, optimal eating may increase life expectancy, as in the “blue zones”, with an impact of health and behavioural factors as well as possible genetic indicators [13]. Ageing-related changes in the gut microbiota may increase chronic inflammation and immune responses leading to degenerative changes and unhealthy ageing [14]. Increased dietary intakes of polyphenols, including anthocyanins, may alter metabolism, chronic syndromes and cell proliferation, possibly related to their antioxidant and anti-inflammatory properties [15]. However, the effectiveness of polyphenols as part of anti-ageing nutrition outside of the “blue zones” has not been proven. The “green diet” emphasising plant foods with minimal amounts of meat such as the Mediterranean diet may increase healthy ageing and also decrease cognitive decline; further, these diets should be more environmentally friendly [16]. Targeted epidemiological studies are important in the design of future clinical trials to evaluate the strategy of healthy diets to extend healthy ageing [17] before these interventions are needed to attenuate the symptoms of the central nervous systems disorders.

The gut microbiota contains more than 10 times the number of cells in the human body. Characterisation of the human microbiota is becoming an important tool for diagnosis, prognosis, risk profiling and precision therapy in humans [18]. The complexity of the healthy microbiota and its relationships to the emergence of disease is related to many factors including patient’s age, lifestyle, ethnicity and diet [18]. Further, molecular characterisation of the microbiota, including viruses and fungi, defines the presence of these microorganisms but does not define their biological activities or their metabolic products. These bioactive metabolites are clearly important in the regulation of the activity of cells throughout the body. A review of 195 meta-analyses with 990 unique health outcomes concluded that the gut microbiota is related to many disease states, including gastrointestinal disease, immune and metabolic outcomes, neurological and psychiatric outcomes, and maternal and infant outcomes [19]. Health interventions that rely on the gut microbiota include prebiotics, probiotics and synbiotics. The potential of these interventions, for example from vegetables and fruit, to modulate host immunity and manage local gastrointestinal and systemic diseases to improve human health has been demonstrated in many clinical trials [20,21]. The bidirectional links between the gut and the brain, the microbiota-gut–brain axis, are modulated by bacterial metabolites, such as tryptophan derivatives, short-chain fatty acids, branched-chain amino acids and peptidoglycans [22]. The gut microbiota has been linked to many chronic conditions of the central nervous system, including autism, anxiety, schizophrenia, Parkinson’s disease and Alzheimer’s disease as further discussed in later sections. In addition, disturbances in the gut microbiota can initiate chronic low-grade inflammation leading to unhealthy ageing [14]. Thus, the central nervous system is a particular focus for new interventions in chronic conditions that leverage the microbiota–gut–brain axis [23].

Nutrition plays an important role in the development of the gut microbiota. Maternal health pre-conception and during pregnancy influences the microbial and cognitive development of the offspring especially during the first 1000 days [24,25]. The Barker hypothesis postulates the foetal origin of adult diseases; recent data propose an important role of PPARs in this process as these receptors are important in the transition of foetus to embryo in mammals [26]. Neuropsychiatric diseases in the adult may be a result of viral or bacterial infections subtly changing foetal brain development [27]. Thus, prevention of adult-onset neuronal disorders may have a component in the prevention of infection and improved nutrition during pregnancy and the first 1000 days.

Another factor in the pathophysiology of major psychiatric disorders such as bipolar disorder, depression, anxiety disorder and schizophrenia is oxidative stress which may be linked to these diseases by activation of a class of calcium channels, thus linking oxidative stress to calcium influx; this could explain the responses to some antioxidants [28]. Inflammation has also been linked to neuronal diseases such as Alzheimer’s disease [29]. The signalling of inflammation across the gut–brain axis is important in the maintenance or normal physiology as well as in the pathology of inflammation-related neuronal damage and disease [30].

Insulin-like growth factor 1 (IGF-1) has been a key player in brain development in younger children [31]. Role of IGF-1 in autism development and suppressing neurologic defects has been observed [31,32,33]. Reduced IGF-1 activity has also been associated with age-related changes such as cognitive decline [34]. The metabolite of IGF-1, cyclic glycine-proline, was found in blackberry anthocyanins; administration increased cyclic glycine-proline in the cerebrospinal fluid of patients with Parkinson’s disease, a condition with IGF-1 deficiency, but no clinical measurements were reported [35]. Thus, IGF-1 could be an important target for improving brain-related disorders.

Anthocyanins or their metabolites may target multiple causes of neuronal disorders, thus an evaluation of their therapeutic potential is warranted. As an example, anthocyanin intake was inversely associated with depressive symptoms in a dose-response manner [36]. Evidence from animal models suggested that these responses of anthocyanins could result from their inhibition of monoamine oxidases and mitochondrial enzymes catalysing oxidation of monoamines [37]. Further, anthocyanins modify the gut microbiota thus providing possible alternative mechanisms for disease management and prevention in the increased cardiovascular and neurodegenerative diseases, cancers and bone loss of the ageing population [38]. However, recommendations for clinical use of anthocyanins are restricted by the low number of clinical trials showing efficacy, their neuroprotective rather than neurorestorative actions, their low oral bioavailability especially to the brain, and possible differences between anthocyanins [39]. Despite these potential limitations, anthocyanins may have therapeutic effectiveness for the disorders of central nervous systems. The following sections will present relevant studies with anthocyanins on central nervous system disorders, highlighting their therapeutic potential.

### 3.1. Autism Spectrum Disorder

Autism spectrum disorders (ASD) include social communication difficulties and repetitive sensory-motor behaviours [40]. This neurodevelopmental disorder is heterogeneous but may involve a range of causes including changes in the gut microbiota, inflammation and oxidative stress. There is limited evidence for the association of environmental factors such as heavy metals, especially inorganic mercury and lead, and vitamin D deficiency with ASD; possible mechanisms include oxidative stress, inflammation, neurotransmitter alterations and changes in signalling pathways [41]. Many factors such as medication exposures and gastrointestinal co-morbidities that change the gut microbiota are present in ASD [42]. Further, transplantation of gut microbiota from humans with ASD into germ-free mice initiated autistic behaviours by production of neuroactive metabolites [43]. In autistic children with constipation, gut microbiota changes have been identified leading to decreased faecal acetate and butyrate but increased valerate concentrations [44]. The importance of the gut microbiota is shown by Microbiota Transfer Treatment as a potential therapy for ASD; in 18 children, follow-up after 2 years showed that improvements in gastro-intestinal symptoms were maintained while further improvements in autism-related symptoms were measured [45]. Prebiotics and synbiotics may produce improvements for some behavioural and gastrointestinal symptoms in ASD but the evidence should be expanded [46,47]. Further, there is evidence for benefits of probiotics in children with ASD showing behavioural and gastrointestinal improvements [48].

Enteroendocrine cells (EC) in the gut produce 95% of the body’s 5-hydroxytryptamine (5-HT, serotonin); chronic exposure to the gut microbiota increases 5-HT synthesis by an increased EC proliferation [49]. Dietary tryptophan is metabolised by the kynurenine pathway (95%) and 5-HT [50]. However, the role of 5-HT in autism is unclear as selective serotonin reuptake inhibitors have modest effects on some repetitive behaviours but do not ameliorate core autism symptoms [51]. The kynurenine pathway may be more relevant with ASD children showing increased concentrations of toxic kynurenine metabolites either causative or resultant from acute and chronic inflammation together with increased anti-inflammatory cytokines [52]. Kynurenine pathway overexpression may be a part of aberrant neurodevelopment in ASD leading to increased neurotoxic metabolites and excitotoxicity [53]. Intestinal-derived vitamins such as the B group vitamins may play a significant role in the function and pathophysiology of the central nervous system including regulation of the tryptophan-kynurenine pathways [54]. Further, dl-leucovorin, a reduced folate, improved some symptoms in children with ASD [55].

Eating disorders are common in children with ASD with dietary changes hard to implement because of tantrums and behavioural problems [56]. Their strong food selectivity alters their gut microbiota with increased short-chain fatty acids and 5-HT-producing bacteria which can then change gastrointestinal function [57]. The maternal diet during gestation as well as the diet of children with ASD may be modifiable risk factors for both the improvement and worsening of symptoms [58]. Prenatal micronutrient supplementation has been proposed as a preventative measure for the development of central nervous system issues including ASD [59]. Vitamin D deficiency during pregnancy and early childhood could impact the developing brain to increase the risk of ASD in children; vitamin D supplementation improved symptoms in children with ASD but the mechanism is unclear [60]. Further nutritional interventions could include the ketogenic diet, defined as a high-fat, appropriate-protein and low-carbohydrate diet, to influence human health [61]. Improvements in autistic behaviour have been reported following intervention with a ketogenic diet, with possible mechanisms including modulation of oxidative stress, neurotransmitters and the gut microbiota [62].

Increased oxidative stress has been proposed as crucially important to the neuroinflammation in ASD suggesting that treatments to decrease concentrations of reactive oxygen species may have therapeutic benefits [63]. Children with ASD may be more vulnerable to oxidative stress and redox imbalance from imbalances in glutathione concentrations and decreased glutathione reserve capacity [64]. Proposed therapies of ASD such as omega-3 fatty acids may lower neuroinflammation by targeting oxidative stress to improve intestinal homeostasis but randomised clinical trials have been inconclusive [65,66].

Although anthocyanins alter the gut microbiota and decrease both inflammation and oxidative stress, no study has reported therapeutic benefits with anthocyanins in people with ASD. However, treatment with an anthocyanin-containing extract from blueberries decreased neuroinflammation and gut inflammation, modulated the gut microbiota and improved serotonin concentrations in the gut and prefrontal cortex to ameliorate autism-like behaviours in a valproic acid mouse model of autism [67]. There are many changes that could improve life outcomes for autistic people [68] and further research on chronic interventions with anthocyanins during pregnancy and in childhood would seem to be justified.

### 3.2. Major Depressive Disorder

Recurrent depressive episodes characterise major depressive disorder, predicted to become the major cause of burden of disease worldwide by 2030 [69]. The proposed mechanisms of the disorders have included the monoamine hypothesis, changes in the hypothalamus–pituitary–adrenal axis, neuroinflammation, neuroplasticity, neurogenesis, changes in brain structure and function, genes and epigenetics, and the role of life events [69]. Neuroinflammation [70] as well as oxidative and nitrosative stress [71] may be key targets for future therapeutic advances for major depressive disorder. Further, the changed gut microbiota-derived short-chain fatty acids and metabolites in major depressive disorders may be a realistic target for interventions such as faecal microbiota transfer and improved dietary health including probiotics [72].

Susceptibility to disease in adults has been linked to changes in intrauterine development. This suggests that optimal nutrition during placental development can minimise adult disease such as metabolic syndrome [73]. This concept has been extended to mental illness where maternal infections producing inflammation could alter foetal brain development leading to neuropsychiatric conditions later in life [27,74]. However, a study using Mendelian randomisation has shown no causal effects between low birth weight and neuropsychiatric conditions including schizophrenia, major depressive disorder and attention-deficit hyperactivity disorder [75]. Further research may indicate whether changes in nutrition for pre-pregnant and pregnant mothers can decrease these neuropsychiatric disorders in their children, in a similar way that treatment with folic acid starting 5–6 months before conception decreased the risk of neural tube defects [76].

### 3.3. Anxiety Disorders and Depression

Anxiety and depressive disorders belong to the internalising disorders and are highly co-morbid [77]. Anxiety disorders, the most common type of mental illness, show excessive fear and anxiety or avoidance of perceived threats [78]. The global prevalence of anxiety disorders increased by 25.6% and of major depressive disorder by 27.6% due to the COVID-19 pandemic in 2020 [79]. Depression describes sad or irritable mood disorders that decrease quality of life. Obesity defined as an increased body mass index predicts chronic anxiety and depression symptoms likely related to prolonged inflammation due to poor dietary lifestyle and inactivity [80]. The high co-morbidity and the role of obesity in human anxiety and depression suggest that changes in the gut microbiota are relevant such as reported higher abundance of pro-inflammatory bacteria and lower abundance of short-chain fatty acid-producing bacteria [81]. Dietary changes associated with changed risk of developing anxiety and depression such as increased omega-3 fatty acid, prebiotic and micronutrient intakes also alter the gut microbiota [82]. Further, gut dysbiosis in rodents caused by stress, high-fat diet or antibiotics caused anxiety- and depression-like behaviours which can be reversed by probiotics; the link between gut dysbiosis, anxiety and depression in rodents is possibly neuroinflammation [83] following dysregulation of microRNAs in the gut and brain [84]. In a mouse model of lipopolysaccharide-induced inflammation, altered neurotransmission in the basal lateral amygdala may produce neuroinflammation-induced anxiety and depressive behaviours [85]. Prebiotics promote anxiolytic and anti-depressive effects in rodent models with plausible mechanisms, but the limitation is that human studies showing clear improvements are scarce [86]. One of the few studies in adult humans showed a link between probiotic-induced changes in the gut microbiota and reduction of stress and anxiety [87]. There is clear evidence of foetal influence as anxiety in 20-year-old offspring has been associated with both maternal and paternal mental health problems [88]. Further, preconception anxiety was related to anxiety-related maternal–infant bonding problems at 12 months after birth which was predicted by anxiety symptoms occurring in young adulthood [89]. These links suggest that extending preconception and prenatal healthcare interventions for the mother for at least the first 12 months after birth will decrease the risk of anxiety disorders in early adulthood of the offspring. Although clinical trials are not available, traditional knowledge attributing anxiolytic and anti-depressive responses to foods containing polyphenols such as anthocyanins may provide treatments for anxiety disorders by improving the gut microbiota [90].

### 3.4. Attention-Deficit Hyperactivity Disorder (ADHD)

ADHD is a lifetime neurodevelopmental condition with diagnosis requiring the presence of six or more symptoms in either the inattentive or hyperactive and impulsive domains, or both, with first-line treatment being psychostimulants such as methylphenidate [91]. The pathophysiology of ADHD is uncertain but the catecholaminergic neurotransmission system may be the major factor, leading to neuronal oxidative stress and inflammation [92]. Recent research has proposed that ADHD patients have a different gut microbial composition which could increase neuronal damage; evidence suggests that omega-3 fatty acids and probiotics may have some therapeutic usefulness in paediatric patients by changing the gut microbiota [92]. In preadolescent children with ADHD defined at age of 10 years, bacterial diversity and composition at age 6 months was associated with symptom development [93]. In children aged 6–16 years with ADHD, decreased plasma TNF-α concentrations negatively correlated with ADHD symptoms may be related to changes in the gut microbiota [94]. Gut microbiota changes in ADHD have been confirmed by meta-analysis [95]. Maternal immune activation triggered by chronic non-resolving inflammation during pregnancy has been proposed as a cause of neurodevelopmental disorders such as ADHD [96]. Micronutrient supplementation, for example with iron and zinc, may possibly provide limited improvements in some parameters [97]. Despite the potential involvement of the gut microbiota, neuronal oxidative stress and inflammation in the pathology of ADHD, there are no studies using anthocyanins to mitigate the condition.

### 3.5. Schizophrenia

Schizophrenia is a severe psychiatric condition showing reality distortion, cognitive impairment, disorganisation and the clinical poverty syndrome with the neurodevelopmental hypothesis from birth cohort studies suggesting that events in utero, at birth or in early life are causes of the adult disease [98]. Perinatal complications such as infections have been strongly implicated as a risk factor for schizophrenia in the offspring but variability in study design and subjects has made interpretation difficult [27]. The complexity of schizophrenia suggests that many biological pathways may be involved, including changes in gene expression, possibly leading to an increased oxidative stress as many signalling and metabolic pathways in the brain increase reactive oxygen species formation and redox imbalance in schizophrenia [99]. Together with oxidative stress, neuroinflammation is a plausible hypothesis for schizophrenia, including cytokines inducing peripheral inflammation interacting with central dopaminergic pathways, microglial activation of central inflammation, neurogenesis as a consequence of neuroinflammation, and the role of acute phase reactants such as C-reactive protein [100]. Dysregulation of inflammatory mediators with increased pro-inflammatory and decreased anti-inflammatory cytokines may increase symptom severity in schizophrenia, possibly related to early childhood trauma and gut microbiota changes [101]. Alterations in the gut microbiota have been linked to the pathogenesis, development, severity and prognosis of schizophrenia with dysbiosis altering the kynurenine–tryptophan pathway to increase the kynurenine pathway and decrease the serotonin pathway of catabolism [102]. However, causality between gut microbiota alterations and psychosis has not been established. The potential role of the gut microbiota in schizophrenia, in particular increased cytokine concentrations, decreased gut membrane and blood–brain barrier integrity, altered neurotransmitters and decreased short-chain fatty acid synthesis, suggests the therapeutic potential of prebiotic/probiotic combinations, in particular to reduce the metabolic alterations during antipsychotic therapy [103]. Further, targeting the gut–microbiota axis with probiotics, prebiotics, antibiotics, or faecal microbiota transplantation may decrease the cognitive impairment as a predictor of negative outcomes in schizophrenia [104]. Despite anthocyanins having antioxidant and anti-inflammatory effects and changing the gut microbiota, no studies on anthocyanins in schizophrenic patients were found.

### 3.6. Alzheimer’s Disease

Alzheimer’s disease is the main cause of dementia, usually in the elderly and associated with increasing amyloid-β inducing the spread of tau pathology [105]. Modifiable risk factors include pre-existing diseases, unhealthy lifestyles and environmental exposures, while active involvement in cognitive activities reduced risk [106]. Physical exercise improves cognitive health in brain ageing and Alzheimer’s disease, possibly by reducing reactive oxygen species and promoting the low concentrations required for optimal cellular function [107]. Oxidative stress is an early clinical feature of Alzheimer’s disease with oxidative modification of macromolecules leading to alterations in function, especially of the mitochondria [108]. Changes in the gut microbiota could increase amyloid-β aggregation, neuroinflammation, oxidative stress and insulin resistance in the brain, suggesting a relationship between gut dysbiosis and the development of Alzheimer’s disease [109]. Gut dysbiosis may lead to the development of local and systemic inflammation since dysbiosis may increase gut permeability to infectious agents such as bacteria and viruses, leading to neuroinflammation and neuronal damage [110]. Microglia as innate immune cells in the brain may be the key players in neuroinflammation in Alzheimer’s disease; potential treatments targeting the microglial priming and responses may be disease-modifying [29]. This role of gut dysbiosis in causing neuroinflammation also suggests that interventions such as prebiotics to stimulate the production of short-chain fatty acids, probiotics and faecal microbial transplantation to improve the gut microbiota may be useful in ameliorating symptoms and the progressive worsening of Alzheimer’s disease [111]. There are strong suggestions that these interventions can become new treatment options to reduce the risk or delay the onset in patients at high risk for Alzheimer’s disease [112]. Trials in humans have been reported but large-scale clinical trials are still essential to understand the potential benefits of alteration of the gut microbiota. Initial clinical studies include the prebiotic, fructan, which reduced the risk of Alzheimer’s disease development [113]. Further, clinical trials with probiotic mixtures in Alzheimer’s disease or mild cognitive impairment showed improvements in cognition with decreased markers for oxidative stress and inflammation [114]. No studies were found investigating faecal microbial transplantation in Alzheimer’s disease patients. While there are no published studies on anthocyanin treatment in Alzheimer’s disease, anthocyanin supplementation with cherry juice or blueberries improved mild cognitive impairment in older patients with mild memory decline [115,116] suggesting that chronic studies with anthocyanins on cognition in schizophrenic patients should be considered.

### 3.7. Parkinson’s Disease

Parkinson’s disease is a neurodegenerative condition usually with a long prodromal period and defined by bradykinesia combined with rest tremor or rigidity or both; no current treatment has been shown to slow or stop progression of the disease [117]. The pathophysiology is complex, but the earliest event of Parkinson’s disease pathogenesis could be a change in the gut microbiota [117]. This altered microbiota may produce toxins that increase production of α-synuclein in the enteric nervous system which may then propagate in a prion-like way to the central nervous system to accelerate Parkinson’s disease pathogenesis [118]. Further, gut dysbiosis could increase gut permeability and general systemic inflammation altering the function of microglia, astrocytes and endothelial cells in the brain to increase neuronal damage and death leading to progression of the disease [119]. In addition, neurotoxins that inhibit mitochondrial complex 1 activity produce neuroinflammation and induce Parkinson’s disease [120]. Oxidative stress is important in the progressive neurodegeneration in Parkinson’s disease. Excessive reactive oxygen species promote cell death pathways such as apoptosis, and cytoplasmic and autophagic cell death suggesting that new therapeutic options should include targeting oxidative stress [121]. Preclinical studies with anthocyanins and their phenolic acid metabolites such as protocatechuic acid have demonstrated antioxidant, anti-inflammatory and anti-apoptotic effects together with prevention of protein polymerisation into plaques and stimulating autophagy that could be effective in treating Parkinson’s disease [122]. However, these promising leads have not yet been translated into therapeutic advances for patients with Parkinson’s disease; problems include the poor oral bioavailability of anthocyanins and limited evidence of neurorestorative properties.

### 3.8. Epilepsy

Epilepsy is characterised by spontaneous seizures with neurobiological, cognitive and psychosocial consequences leading to increased morbidity, disability and mortality [123]. Although a wide range of pharmacological treatments are available, around 30% of epileptics remain drug resistant. Gut dysbiosis is involved in the development and susceptibility of adults to epilepsy as this is associated with neuroinflammation, altered neuromodulators and disruption of the blood–brain barrier [124]. This relationship is strengthened by decreased symptoms with antibiotics, probiotics, the ketogenic diet and faecal microbial transplantation [124,125]. Further, the ketogenic diet, a high-fat, low-carbohydrate diet that results in ketosis, effective in about one-third of drug-resistant epilepsies, produces anti-inflammatory responses with likely neuroinflammatory pathways including adenosine modulation, ketone bodies, mTOR pathways, PPARγ, NLRP3 inflammasome and gut microbiota [126]. Pro-inflammatory cytokines such as IL-1β, IL-6, TNF-α and IFN-γ are increased in animal models of epilepsy and in human studies [127]. Together with neuroinflammation, oxidative and nitrosative stress is rapidly induced in animal models of epilepsy; anti-inflammatory and antioxidant drugs may moderate disease severity by providing neuroprotection and decreased cognitive deficits in preclinical studies and in adults with drug-resistant epilepsies [128]. Antioxidants may provide neuroprotection by targeting mitochondrial oxidative stress to modify apoptosis–autophagy and autophagy–ferroptosis crosstalk [129]. However, no studies have specifically tested the role of anthocyanins in epilepsy.

## 4. Conclusions

Central nervous system disorders show different ranges of symptoms that define the disease state. Common changes in these disorders, present to varying extents, include foetal origin, development during childhood, changes in the gut microbiota, and increased oxidative stress and neuroinflammation leading to nerve damage. Mechanisms of action of anthocyanins in chronic human disease include decreased oxidative stress and inflammation and changes in the gut microbiota. The broad commonalities between symptoms of central nervous system disorders and the mechanisms of therapeutic actions of the anthocyanins infer that anthocyanins are potential treatments for these disorders. While clinical evidence is limited, the information presented in this review forms the basis for in-depth evaluation of the role of chronic treatment with anthocyanins as adjunctive therapy for central nervous system disorders. Further, initiation of treatment as early as possible, possibly even pre-conception and continued throughout pregnancy, the first 1000 days, childhood and early adulthood may reduce the risk of central nervous system disorders in adults.

## Data Availability

Not applicable.
